# Adjuvant chemotherapy in T1a/bN0 breast cancer with a high 21-gene recurrence score (> 25): a 10-year follow-up in a real-world cohort

**DOI:** 10.1007/s12282-024-01652-9

**Published:** 2024-11-27

**Authors:** Daniela Katz, Ilan Feldhamer, Yael Wolff-Sagy, Hadar Goldvaser, Ariel Hammerman, Daniel A. Goldstein

**Affiliations:** 1 The Helmsley Cancer Center, Shaare-Zedek Medical Center, Faculty of Medicine, The Hebrew University Jerusalem, 12 Beyth St, 9103102 Jerusalem, Israel; 2 Breast Medicine Service, Department of Medicine, Memorial Sloan Kettering Cancer Center, New York, USA; 3 Clalit Health Services, Tel-Aviv, Israel; 4 Medison Pharma Ltd, Petah Tikva, Israel; 5 Davidoff Cancer Center, Rabin Medical Center, Petah Tikva, Israel; 6 Faculty of Medicine, Tel Aviv University, Tel Aviv, Israel

**Keywords:** Adjuvant, Breast cancer, Chemotherapy, OncotypeDX^©^, T1/bN0

## Abstract

**Background:**

In ER + /HER2- early breast cancer (BC), 21-Gene Recurrence Score (RS) > 25 indicates high-risk of distant-recurrence and predicts benefit from adjuvant chemotherapy (aCT) regardless of tumor-size. However, T1a/b (≤ 1 cm) node-negative (N0) tumors, regarded as of low risk of recurrence, were under-represented in the RS trials. We therefore aimed to investigate the benefit of aCT in patients with T1a/bN0 BC, RS > 25, where clinical and genomic risk indicators are discordant.

**Methods:**

This retrospective observational cohort study utilized Israel’s national Oncotest database to identify Clalit Health Services (CHS) members, diagnosed with T1a/bN0 HR + /HER2- BC, who underwent RS testing between February 2006, and December 2019. Patients with RS > 25 who received aCT were matched 1:1 by propensity-scoring to similar patients receiving no aCT. Invasive disease-free survival (iDFS) and distant recurrence were the study endpoints. Patient demographic and clinical data were obtained from CHS’s centralized database. Kaplan––Meier analysis with log-rank testing was used for comparing outcomes.

**Results:**

During the study period, high-risk RS result (> 25) was identified in 156/9858 patients of the study cohort. aCT was administered to 74 (47.4%) and median follow-up was 121 months. Within the 148 matched-cases, eighteen iDFS-events occurred, nine (12.1%) in each group (χ^2^ = 0.72, p = 0.39). Four (5.4%) of the aCT treated and three (4.0%) of the untreated patients were diagnosed with distant recurrence (χ^2^ = 0.22, *p* = 0.64).

**Conclusions:**

In this study cohort, patients with T1a/bN0 BC, RS > 25 that received aCT, did not have improved outcomes and the 21-Gene RS > 25 was not found to be predictive, possibly due to the low number of events observed.

## Introduction

The OncotypeDX^®^ 21- gene expression assay (Genomic health, Redwood City, CA) is prognostic and predictive of benefit from adjuvant chemotherapy (aCT) for women with hormone receptor-positive (HR +) and human epidermal growth factor receptor 2 negative (HER2-) early breast cancer (BC) [1–3].

Recurrence score (RS) > 25 was established as a threshold for benefit from aCT based on the TAILORx study [3, 4], which examined the benefit of aCT in lymph node negative (N0) BC. Originally high-risk RS was defined as RS ≥ 31, however, to minimize the potential of undertreatment the high-risk threshold was adjusted to RS > 25 [3, 4].

With the increased use of screening mammography, approximately 20% of newly diagnosed breast tumors are in the T1a/b (tumor size < 0.5–1 cm) N0 stage [5]. Discrepancy between traditional clinicopathological parameters associated with clinical low risk (e.g. T1a/b, N0), and genomic high-risk as determined by RS > 25, raises a treatment dilemma. Prospective data on outcomes of the subgroup of patients with T1b (> 0.5-1cm) N0 and RS > 25 who received aCT is scarce. Even the TAILORx study, that included 188 patients with T1b tumors and RS > 25 the benefit of aCT among patients who received and did not receive aCT (180 vs 8 patients, respectively) was not reported separately [2].

In the absence of prospective data on the benefit of aCT in patients with T1a/bN0 BC and RS > 25, we aimed to examine in a retrospective real-world cohort, whether aCT added to adjuvant endocrine therapy (ET) improved outcomes in such patients. To our knowledge this is the largest study to examine this question.

## Methods

This retrospective observational cohort-study utilized Israel’s national Oncotest database, which contains RS-results from the OncotypeDX^®^ 21- gene expression assay, histology subtype, grade, tumor size and HR status of the tested tumors. The Oncotest database includes data of all women in Israel diagnosed with ER/PR positive HER2 negative invasive breast cancer, for whom their oncologist opted to perform the OncotypeDX^®^ 21- gene expression assay.

Within the Oncotest database, we identified women with T1a/bN0 HR + /HER2- BC, who underwent RS testing between February 1, 2006, and December 31, 2019, and were members of Clalit Health Services (CHS), that covers about half of the total Israeli population. Data on aCT treatments provided to patients with RS > 25, the clinical outcomes, demographic characteristics, clinicopathological features and patients’ comorbidities were all extracted from CHS centralized electronic database.

Providing aCT is not standardized and is administrated according to the treating physician’s discretion. ET was recommended to all patients and provision of drug to patients was recorded. Patients with who received aCT were matched 1:1 by propensity-scoring to similar patients receiving no aCT. The primary study endpoint was the occurrence of an invasive disease-free survival (iDFS) event, as defined within the TAILORx [4]; The secondary endpoint was a distant-recurrence event. The outcome data lock was set on June 30, 2023, ensuring a minimum follow-up interval of at least 4.5 years.

## Statistical analysis

Descriptive statistics was used for the sociodemographic and clinicopathologic characteristics. All patients’ characteristics were included as categorical variables except for age. To calculate propensity score (PS) for matching non-aCT to aCT patients, a logistic regression was executed with age group, socioeconomic status [6], Charlson-comorbidity index [7], tumor size (≤ 0.5 vs. > 0.5–1 cm), grade, HR status, histology and oncotype score category (26–30 vs. > 30) as independent variables. Of notice, a comparison between the oncotype score categories was conducted to identify an increased benefit of chemotherapy at a higher RS threshold, using the original threshold for chemotherapy benefit (RS ≥ 31) versus the currently accepted threshold (RS > 25) (1, 3). PS with nearest neighbor with replacements and with a caliper of 0.25 was conducted to match patients with aCT with non-aCT on a 1:1 ratio. Of the non-aCT patients, 20 (27%) were used as controls once, 11 (15%) were used as controls with two repetitions, and 7 (9%) were used as controls with three to six repetitions.

Mean-times to an iDFS and distant-recurrence events were compared by Kaplan–Meier analysis with log-rank testing. Patients were censored at data lock date. All tests were 2-sided and *p* ≤ 0.05 was considered statistically significant. Descriptive statistics, PS calculation and matching and Kaplan–Meier analysis were conducted by SPSS software version 29 (IBM). Kaplan–Meier survival curves were created with R statistical software version 3.5.0 (R Foundation).

The study was approved by the CHS Community Helsinki ethics committee (approval 0074–22-COM2) and was exempt from the requirement to obtain informed consent owing to the retrospective design.

## Results

**Fig. 1:**
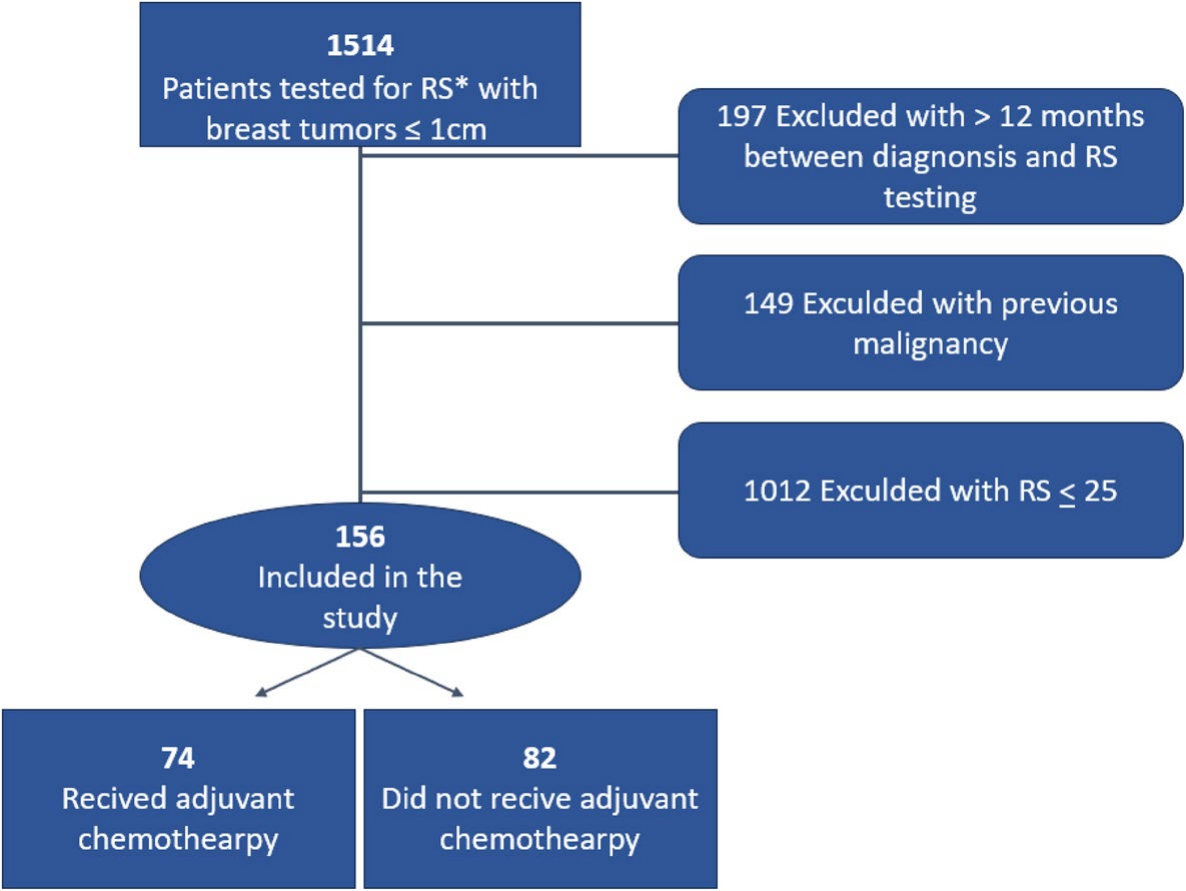
Consort flow diagram. The patient cohort was extracted from the Oncotest database, which includes RS results from OncotypeDX^®^ 21- gene expression assay, histology subtype, grade tumor size and hormone receptor status. *RS: OncotypeDX^®^ 21- gene recurrence score

A total of 156 patients met the inclusion and exclusion criteria (Fig. 1), almost half (74 patients, 47.4%) received aCT (Table 1). Median duration of the study follow-up was 121.0 months (95% CI, 108.4–133.6). Patients who received aCT were younger, had less comorbidities, and had a higher RS (Table 1), type of aCT received is shown in Table 2. Duration of ET was not significantly different between both groups.

**Table 1:** Demographic and clinicopathological characteristics of patients by adjuvant chemotherapy treatment before propensity-core matching and after

		Non-matched			Propensity score-matched		
		no aCT	aCT	p-value	no aCT	aCT	p-value
		n = 82	n = 74		n = 74	n = 74	
Sociodemographic							
Age	24–49	8 (9.8%)	19 (25.7%)		16 (21.6%)	19 (25.7%)	
	50–59	24 (29.3%)	22 (29.7%)		20 (27%)	22 (29.7%)	
	60–69	35 (42.7%)	27 (36.5%)		33 (44.6%)	27 (36.5%)	
	70–74	15 (18.3%)	6 (8.1%)	0.28	5 (6.8%)	6 (8.1%)	0.79
	median	62.5 (54–8.3)	57.5 (49–4.3)	0.002	60 (51–65.3)	57.5 (49–64.3)	0.38
SES* (6)	low	11 (13.4%)	19 (25.7%)		19 (25.7%)	19 (25.7%)	
	medium	40 (48.8%)	30 (40.5%)		32 (43.2%)	30 (40.5%)	
	high	31 (37.8%)	25 (33.8%)	0.15	23 (31.1%)	25 (33.8%)	0.93
Clinical pathological properties							
Tumor size (cm)	</= 0.5	6 (7.3%)	2 (2.7%)		5 (6.8%)	2 (2.7%)	
	>0.5–1	76 (92.7%)	72 (97.3%)	0.19	69 (93.2%)	72 (97.3%)	0.24
Grade	1	8 (9.8%)	3 (4.1%)		5 (6.8%)	3 (4.1%)	
	2	44 (53.7%)	37 (50%)		48 (64.9%)	37 (50%)	
	3	19 (23.2%)	25 (33.8%)		16 (21.6%)	25 (33.8%)	
	Unknow	11 (13.4%)	9 (12.2%)	0.32	5 (6.8%)	9 (12.2%)	0.17
Histology	IDC/Unknown	73 (89%)	71 (95.9%)		70 (94.6%)	71 (95.9%)	
	ILC	9 (11%)	3 (4.1%)	0.1	4 (5.4%)	3 (4.1%)	0.7
ER^#^	0–1	7 (8.5%)	5 (6.8%)		9 (12.2%)	5 (6.8%)	
	2–3	75 (91.5%)	69 (93.2%)	0.67	65 (87.8%)	69 (93.2%)	0.26
PR^^^	0–1	56 (69.1%)	51 (68.9%)		51 (68.9%)	51 (68.9%)	
	2–3	25 (30.9%)	23 (31.1%)	0.97	23 (31.1%)	23 (31.1%)	1.000
OncotypeDX recurrence score	26–30	63 (76.8%)	25 (33.8%)		29 (39.2%)	25 (33.8%)	
	31 +	19 (23.2%)	49 (66.2%)	< 0.001	45 (60.8%)	49 (66.2%)	0.5
Charlson comorbidity score (7)	0–1	40 (48.8%)	52 (70.3%)		49 (66.2%)	52 (70.3%)	
	2 +	42 (51.2%)	22 (29.7%)	0.006	25 (33.8%)	22 (29.7%)	0.6

*SES socio-economic status, ^#^*ER* estrogen receptor, ^^^*PR* progesterone receptor

**Table 2:** Adjuvant chemotherapy treatments

	n (%)
IV Doxorubicin & Cyclophosfamide	16 (21%)
IV Doxorubicin & Cyclophosfamide and Taxane	24 (32%)
IV Docetaxel & Cyclophosfamide	33 (45%)
IV Cyclophosfamide Methotrexate 5-FluoroUracil	1 (1%)
Total	74

PS-matching resulted in 74 matched cases. The PS-matched groups demonstrated similar socio-demographic and clinical characteristics (Table 1). In addition, a similar distribution of the PS was observed.

Eighteen iDFS events occurred in the matched cohorts, nine in each group (p = 0.39). A total of seven (4.5%) patients were diagnosed with distant-recurrences, four (5.4%) in the aCT group and three (4%) in the non-aCT group (p = 0.64).

**Fig. 2:**
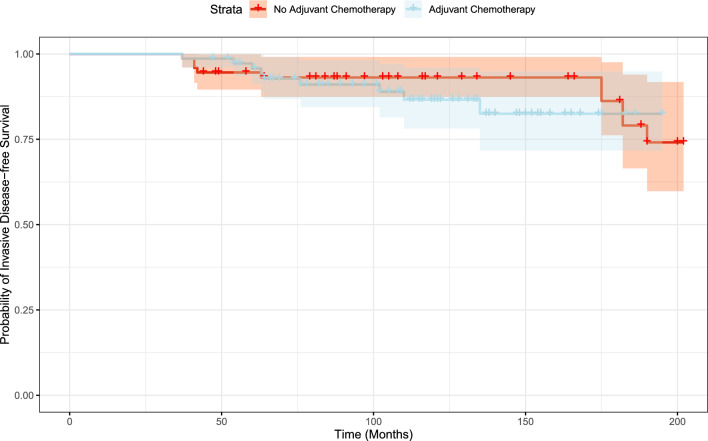
Probability of invasive disease-free survival over time. The shaded area represents the confidence intervals

**Fig. 3:**
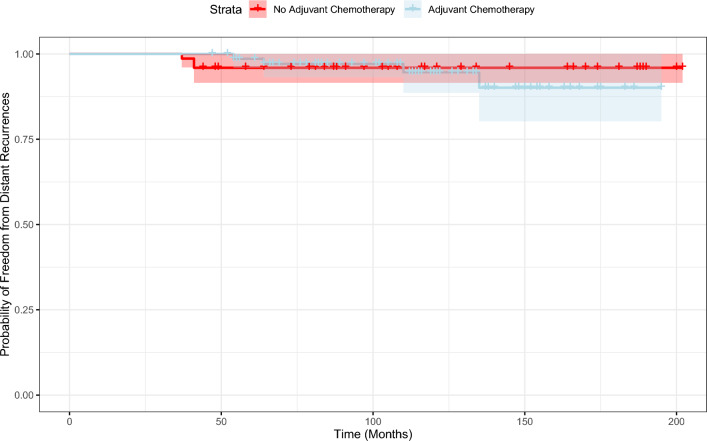
Probability of distant recurrence over time. The shaded area represents the confidence intervals

The mean time to an iDFS event was 171.5 months (95% CI, 160.9–182.1) and 177.6 months (95% CI, 169.2–186.0) in the aCT and non-aCT cohorts, respectively; *p* = 0.4. The mean time to distant recurrence was 186.2 months (95% CI, 178.1–194.5) and 188.7 months (95% CI, 181.7–195.7) in the aCT and the non-aCT cohorts, respectively (p = 0.64). Figure. 2 demonstrates iDFS over time and Fig. 3 the distant recurrence events over time, supporting the good outcome of patients with small tumors N0 BC.

## Discussion

In our real-life retrospective cohort, with a median follow-up of 10-years, patients with T1a/bN0 BC and RS > 25, did not show improved iDFS with aCT compared to those who did not receive aCT. To our knowledge, this is the largest cohort examining this question [2, 8, 9].

We chose RS > 25 as the threshold for benefit from chemotherapy in patients without lymph node involvement based on TAILORx results [4], and the NSABP B-20 results when HER2 positive patients were excluded [3].

In our patient cohort three parameters were associated with aCT administration: age, low Charlson’s comorbidity score and RS ≥ 31. This represents the preferences of Oncologists in real-life when considering aCT in T1a/bN0 BC.

Although approximately 20% of newly diagnosed breast tumors are in the T1a/bN0 stage [5], prospective data on the benefits of aCT in this subgroup is lacking. Perhaps, due to limited adoption of aCT within this subgroup as reflected in the recent St. Gallen recommendations where approximately 90% of expert panelists [10] voted against providing aCT for tumors < 0.8 cm N0, despite a high genomic assay result. Interestingly, same experts were more inclined (up to 40%) recommending aCT for slightly larger, 0.8-1cm tumors.

In our cohort, there were 12.2% iDFS events and 4.5% distant recurrences events. Our findings are consistent with the MINDACT study that showed excellent disease-free survival and distant metastasis-free survival in patients with breast cancer of low-clinical (tumors ≤ 1cm N0) and high-genomic risk who did not receive aCT [11, 12].

Interestingly, we noticed that the mean time to an iDFS or to a distant recurrence event exceeded 171.5 and 186.2 months (Figs. 2, 3), respectively, emphasizing the inherent risk for late-recurrence of ER + /HER2- small tumors which is not predicted by the current RS assay [13].

The strength of our study lies in the highly selective study population, consisting of small N0 tumors with high genomic risk and the lengthy follow-up duration (median of ten years). To our knowledge, this is the largest breast cancer real-life cohort that examined benefit from aCT in T1a/bN0 BC. Very small tumors are usually under-represented in clinical trials, e.g. in the TAILORx study only 13% of patients had tumors 1cm or smaller (tumor 0.5 > were excluded) [4] and in the NSABP B-20 trial (excluding HER2 positive by RT-PCR) only 14.9% had such tumors [3].

In addition, we used the CHS registry, a well-established registry reflecting real- world experience that was used as a validation cohort to develop the RSclin tool, which aimed to individualize prognosis and prediction of chemotherapy benefit [13]. The registry served as a basis for many published BC manuscripts [14, 15].

Our study also has several limitations, primarily its retrospective and uncontrolled nature, leading to an imbalance between characteristics of patients who received aCT and those who did not. We addressed the imbalance using PS matching; however, a few control subjects were matched three to six times, even after applying a relatively high caliper of 0.25 for the PS matching. Another significant limitation is the low number of events observed, consistent with the prognosis of patients with low-clinical risk and high-genomic risk in the MINDACT trial [11].

Another study constraint arises from the selection bias inherent in the study cohort, comprised of patients selected by their treating physician to undergo 21- gene expression assay testing. Ordering genomic testing in Israel is at the discretion of the breast surgeon or treating oncologist and not limited by tumor size. Therefore, the study cohort may not represent the entire T1a/bN0 population and represents patients to whom aCT was considered, younger patients or those with a more luminal B-like phenotype BC (defined by grade 2–3, lower progesterone receptor expression and a higher cell proliferation) associated with an inferior outcome. Evidence for poorer outcomes in patients with histological grade 3 tumors and RS > 25 is provided by the study conducted by Stemmer et al., (13) which examined 10-year outcome of patients with N0 BC based on their RS-result in a comparable cohort.

We believe this limitation did not adversely affect the study results, because the absence of benefit from aCT in these higher-risk tumors would probably extend to a lack of benefit in lower clinical-risk tumors with a more luminal A-like phenotype.

Another limitation is the small subgroup of T1a tumors (8/156 patients), especially those treated with aCT (2/8 patients) prevent drawing conclusions regarding any benefit from aCT in this subgroup.

In summary, within our retrospective cohort, providing aCT did not improve outcomes of patients with T1a/bN0 HR + /HER2- BC with RS > 25, suggesting that RS > 25 was not predictive of benefit from aCT in our study. The low number of events observed in our study supports the good prognosis of these patients.

Prospective trials are still needed to identify at diagnosis those few patients who later experience distant recurrence, possibly by evaluating RS ≥ 31 threshold. Another possibility is integrating clinicopathologic biomarkers with the RS, that could potentially lead to more accurate identification of patients with small tumors at high- risk for recurrence, allowing others to avoid unnecessary aCT. This becomes increasingly important as more patients are diagnosed with T1a/bN0 tumors through routine BC screening.

## References

[1] Paik S, Tang G, Shak S, et al. Gene expression and benefit of chemotherapy in women with node-negative, estrogen receptor-positive breast cancer. J Clin Oncol. 2006;24:3726–34. 16720680 10.1200/JCO.2005.04.7985

[2] Sparano JA, Gray RJ, Makower DF, et al. Clinical outcomes in early breast cancer with a high 21-gene recurrence score of 26 to 100 assigned to adjuvant chemotherapy plus endocrine therapy: a secondary analysis of the TAILORx randomized clinical trial. JAMA Oncol. 2020;6:367–74. 31566680 10.1001/jamaoncol.2019.4794 PMC6777230

[3] Geyer CE Jr, Tang G, Mamounas EP, Rastogi P, Paik S, Shak S, Baehner FL, Crager M, Wickerham DL, Costantino JP, Wolmark N. 21-Gene assay as predictor of chemotherapy benefit in HER2-negative breast cancer. NPJ Breast Cancer. 2018;4:37. 30456299 10.1038/s41523-018-0090-6 PMC6235896

[4] Sparano JA, Paik S. Development of the 21-gene assay and its application in clinical practice and clinical trials. J Clin Oncol. 2008;26:721–8. 18258979 10.1200/JCO.2007.15.1068

[5] Welch HG, Prorok PC, O’Malley AJ, et al. Breast-cancer tumor size, overdiagnosis, and mammography screening effectiveness. N Engl J Med. 2016;375:1438–47. 27732805 10.1056/NEJMoa1600249

[6] Arbel R, Wolff Sagy Y, Hoshen M, et al. Nirmatrelvir use and severe Covid-19 outcomes during the omicron surge. N Engl J Med. 2022;387:790–8. 36001529 10.1056/NEJMoa2204919 PMC9454652

[7] Charlson ME, Pompei P, Ales KL, MacKenzie CR. A new method of classifying prognostic comorbidity in longitudinal studies: development and validation. J Chronic Dis. 1987;40:373–83. 3558716 10.1016/0021-9681(87)90171-8

[8] Pomponio M, Keele L, Hilt E, et al. Impact of 21-gene expression assay on clinical outcomes in node-negative ≤ T1b breast cancer. Ann Surg Oncol. 2020;27:1671–8. 31686348 10.1245/s10434-019-08028-w

[9] Nguyen TTA, Postlewait LM, Zhang C, et al. Utility of Oncotype DX score in clinical management for T1 estrogen receptor positive, HER2 negative, and lymph node negative breast cancer. Breast Cancer Res Treat. 2022;192:509–16. 35084624 10.1007/s10549-022-06530-6

[10] Curigliano G, Burstein HJ, Gnant M, et al. Understanding breast cancer complexity to improve patient outcomes: The St Gallen International Consensus Conference for the Primary Therapy of Individuals with Early Breast Cancer 2023. Ann Oncol. 2023;34:970–86. 37683978 10.1016/j.annonc.2023.08.017

[11] Cardoso F, Lauravan’tVeer J, Bogaerts J, et al. MINDACT Investigators. 70-Gene signature as an aid to treatment decisions in early-stage breast cancer. N Engl J Med. 2016;375:717–29. 27557300 10.1056/NEJMoa1602253

[12] Piccart M, van’tVeer LJ, Poncet C, et al. 70-gene signature as an aid for treatment decisions in early breast cancer: updated results of the phase 3 randomized MINDACT trial with an exploratory analysis by age. Lancet Oncol. 2021;22:476–88. 33721561 10.1016/S1470-2045(21)00007-3

[13] Stemmer SM, Steiner M, Rizel S, et al. Ten-year clinical outcomes in N0 ER+ breast cancer patients with Recurrence Score-guided therapy. NPJ Breast Cancer. 2019;5:41. 31728408 10.1038/s41523-019-0137-3 PMC6841708

[14] Sparano JA, Crager MR, Tang G, et al. Development and validation of a tool integrating the 21-gene recurrence score and clinical-pathological features to individualize prognosis and prediction of chemotherapy benefit in early breast cancer. J Clin Oncol. 2021;39:557–64. 33306425 10.1200/JCO.20.03007 PMC8078482

[15] Rotem O, Peretz I, Leviov M, et al. Clinical outcomes in estrogen receptor-positive early-stage breast cancer patients with Recurrence Score 26–30: observational real-world cohort study. NPJ Breast Cancer. 2023;9:49. 37268607 10.1038/s41523-023-00549-8 PMC10238504

